# How can policymakers incorporate uncertainty (as modelled through foresight) into policy evaluation?

**DOI:** 10.1093/eurpub/ckac129.123

**Published:** 2022-10-25

**Authors:** CA Bana e Costa, MD Oliveira, TC Rodrigues, A Vieira

**Affiliations:** CEG-IST, Instituto Superior Técnico, Technical University of Lisbon, Lisbon, Portugal

## Abstract

**Background:**

Using a robust policy evaluation framework is critical to inform policymakers about the relevance of a policy in a structured way, clarifying its implications and providing arguments and criteria to compare competing options and decide which policy should be prioritised. In the current context of complex societal, environmental and public health challenges, decision-makers demand tools that allow a comparative assessment of the effectiveness of multisectoral policies and their comparison under different scenarios.

**Methods:**

This presentation will detail a socio-technical “desirability-doability” framework (2xD). In 2xD, an additive value model is constructed to measure the desirability of public policies, and their doability is appraised under two contrasted scenarios. For these purposes, the MACBETH approach is used in developing the three group modelling phases of 2xD: (I) Structuring facilitated workshops, (II) Evaluation decision conference, and (III) Desirability-doability decision conference. Tailor-made interactive protocols or questioning procedures are used to elicit group judgements, based on which objective-specific value scores are assigned to the policies, and the objectives are weighted (in II). Finally, we elicit doability scores for the policies under each scenario (in III).

**Results:**

We present how the 2xD framework was applied in Lisbon's urban health policymaking setting and discuss case insights regarding the role of scenario analysis in the appraisal and selection of policies.

**Conclusions:**

The 2xD framework advances knowledge on how to assist policymakers in evaluating and selecting policies and contributes to the literature on overall policy formulation and evaluation. Specifically, the framework enabled a group of policymakers to balance (multicriteria) desirability versus doability of policies under the light of two contrasting scenarios, therefore incorporating uncertainty in their decision-making process.

